# Study on the relationship between PTSD and academic control and academic emotion in primary and middle school students after flood disaster

**DOI:** 10.3389/fpsyg.2024.1429238

**Published:** 2024-08-07

**Authors:** Lili Zhao, Haiyan Wang, Kangning Wang, Chengxuan Shen, Mingda Tao

**Affiliations:** Normal College, Qingdao University, Qingdao, China

**Keywords:** flooding, PTSD, perceived academic control, academic emotions, relationship study, control-value theory

## Abstract

**Purpose:**

To explore the relationship between post-traumatic stress disorder (PTSD) and students’ academic control and academic emotion in the aftermath of a flood disaster. The findings will offer educators and relevant departments valuable insights to understand and facilitate the restoration of learning capabilities among students affected by the disaster.

**Methods:**

This study employed a combined approach of questionnaire surveys and longitudinal tracking. Students from Guangling Primary and Secondary School (Shouguang City, Weifang, Shandong Province) participated in surveys administered in September 2018, December 2018, and September 2019. The instruments utilized included the Post-Disaster Trauma Assessment Questionnaire, the Adolescent Academic Control Scale, and the mathematical version of the Achievement Emotions Questionnaire. Data analysis involved two-factor correlation and mediation effect testing.

**Results:**

Significant differences were observed in overall PTSD scores and its three dimensions between the 1-week and 1-year post-disaster assessments. Both the average PTSD score and the detection rate were higher 1 year after the disaster compared to the first week. Students’ academic control demonstrated a strong positive correlation with positive academic emotions and a significant negative correlation with anxiety-related academic emotions. Cross-lagged regression analysis indicated a predictive relationship: academic control measured 3 months post-disaster significantly predicted academic emotions at the 9-month assessment, and conversely, academic emotions at the 3-month point were predictive of academic control at 9 months. In addition, academic control appears to play a complete mediating role in the relationship between PTSD and academic emotions.

**Conclusion:**

Students exhibited a range of PTSD symptoms following the disaster, with a higher prevalence noted in the first year compared to the initial week. PTSD negatively affects academic standing in these students, and is predictive of both their sense of academic control and their emotional responses to learning. Crucially, academic control and academic emotions exhibit a strong correlation and can mutually affect one another. Interventions aimed at reducing PTSD symptoms, cultivating positive academic emotions, and strengthening students’ sense of academic control must therefore consider the relationship between these factors. This holistic approach will enhance psychological well-being and improve academic performance.

## 1 Introduction

Characterized by its expansive and diverse topography, China is prone to devastating floods and droughts, with events exceeding 10,000 fatalities occurring roughly every 15 years (Xu, 2019). The occurrence of such natural disasters extend far beyond immediate socioeconomic consequences, casting a long shadow on the psychological well-being of both adults and students. Post-traumatic stress disorder (PTSD), a chronic mental health condition, arises from exposure to extraordinarily threatening or catastrophic events (Arnberg et al., 2013). It’s a prevalent and emblematic negative psychological outcome, frequently occurs in the aftermath of disasters (Arnberg et al., 2013). Research indicates that PTSD symptomology is associated with increased substance abuse, sleep disturbances, reduced quality of life and subjective well-being, and a more pessimistic outlook on life satisfaction, collectively emphasizing the significant and negative effect on the physical and mental health of trauma survivors (Karatzias et al., 2013; Zhao et al., 2013; Wang et al., 2018). In addition to their impact on People’s Daily life (Usher et al., 2020), It also leads to a series of negative psychological manifestations, such as fear (Broche-Pérez et al., 2023), anxiety, depression, and increased stress levels (Rodríguez-Fernández et al., 2021).

Adolescents, a particularly vulnerable population, often grapple with a multitude of concurrent psychological issues when confronting post-disaster trauma, including reduced self-perception, strained interpersonal relationships, and a sense of detachment from their values (Rao and Huang, 2020). Studies assessing the prevalence of PTSD among adolescents following the Wenchuan earthquake indicated a rate of 40.1% (Jin et al., 2014), with some research indicating a sustained prevalence of 29.6% even three years post-earthquake (Pan et al., 2015). The effect of Typhoon Wenbya on Shouguang City in August 2018 further exemplifies the potential consequences of natural disasters on the physical and mental health of primary and secondary school students.

Human achievement is significantly affected by emotions, in educational settings, emotions exert a significant effect on students’ cognitive processes, motivation, and overall interest in learning. Academic emotions are emotions that are directly related to school learning, classroom teaching, and academic achievement (Pekrun et al., 2002). These emotions are reflected in students’ responses to academic successes and failures, as well as their emotional states during classroom learning, daily homework completion, and study periods (Yu and Dong, 2005). Positive emotions cultivate a sustained state of positive affect, leading to enhanced learning efficiency and encouraging the adoption of more flexible and creative learning approaches (Ainley et al., 2005). Conversely, negative emotions (e.g., boredom and disappointment, etc.) can reduce learning motivation and effort. Emerging research exploring the relationship between emotions and PTSD highlights the various mechanisms influencing academic emotions.

German scholars Pekrun et al. (2002) proposed the Control-Value Theory of achievement emotions, a framework for analyzing the antecedents (external environment) and consequences (academic performance) of emotions experienced by students. This model hypothesizes that academic control is a crucial prerequisite for the generation and development of academic emotions, exerting a significant effect on academic achievement. While research on emotions in educational contexts was largely overlooked prior to the 21st century, scholars in the fields of teaching, learning, and classroom motivational research currently recognize the indispensable role of emotional factors.

Perceived academic control represents the degree to which students believe they can affect their academic performance, forming a stable psychological factor in the education. This belief reflects students’ confidence in predicting their academic success or failure. As such, perceived academic control plays a critical role in shaping students’ academic emotions and achievement levels (Pekrun et al., 2002). It also significantly affects their decision-making processes regarding academic effort and engagement (Perry et al., 2005). Academic burnout (i.e., emotional exhaustion) has been identified as an important risk factor for the development of PTSD symptoms (Tomaszek and Muchacka-Cymerman, 2022). Tian et al. (2023) showed that self-control can act as an intermediary to indirectly predict academic burnout.

Control-value theory suggests that academic emotions primarily arise from students’ perceived control over tasks and outcomes, alongside their subjective assessment of the activity’s value. The control assessment comprises a student’s evaluation of their own capability to succeed in academic tasks and master course content, with self-efficacy being a paramount factor (Pekrun, 2006). The theory proposes that the mediating factors regulating the influence between emotions and the social environment are the control and cognitive assessment of value, and that the influence produced by the environment and emotions is bidirection.

Perry et al. (2001) identified a significant effect of academic control on students’ emotional experiences, thus impacting academic performance. Students exhibiting higher levels of academic control tend to invest greater effort in learning, experiencing reduced anxiety and enhanced academic performance. Further analysis by Ruthig et al. (2008) studied the interaction of academic control and emotions on college student performance. Their findings indicated boredom and anxiety as negative predictors of academic success, with a significant interaction effect. Specifically, students possessing high academic control, yet experiencing higher levels of boredom and anxiety, demonstrated lower average credit scores. Conversely, this interaction was not significant for students with lower academic control.

Ruthig et al. (2009) noted that optimism and social support serve as protective factors against stress and depression, with academic control acting as a mediating variable. Essentially, academic control mediates the protective effects of optimism and social support on mental well-being. Complementing this research, Yao et al. (2010) established a significant negative correlation between academic self-efficacy and test anxiety, suggesting self-efficacy as a predictor of reduced test anxiety.

Xin et al. (2018) evaluated the coordination between emotional motivation intensities, working memory, and self-control. Their findings suggest that optimal coordination between emotional and cognitive systems occurs under specific conditions: language working memory under low approach motivation positive emotions and spatial working memory under low avoidance motivation negative emotions. This coordination minimizes cognitive resource depletion and enhances self-control. Supporting this notion, Cheung et al. (2014) proposed that individuals exhibiting higher self-control tend to prioritize resisting temptation, resulting in higher positive emotions and reduced negative emotions. Moreover, Tangney et al. (2004) demonstrated a positive relationship between the implementation of self-control and the experience of positive emotions.

Significant life events possess the capacity to challenge an individual’s established mental models concerning both self-perception and the external world, prompting a reconstruction of thoughts regarding past experiences and unknown circumstances. Individuals who have endured trauma often engage in repeated recognition and reflection upon the traumatic events, influencing both their physical and psychological responses. While numerous theoretical frameworks have concentrated on analyzing the effect of cognitive factors on post-traumatic psychological responses, the role of emotional factors, particularly positive emotions, should not be underestimated. The positive emotion expansion-construct theory hypothesizes that positive emotions broaden an individual’s thought processes, enhance behavioral flexibility, and facilitate the development of lasting physiological, psychological, and social resources. This contributes to the mitigation of negative post-traumatic psychological sequelae and cultivates healthy individual growth (Nelson, 2009).

Despite the abundance of research exploring emotion theories associated with academics, theoretical frameworks specifically focused on academic emotions remain relatively limited. In addition, analyses into the effect of post-disaster psychological environments on academic performance are notably limited, with even less exploration of the predictive relationship between post-disaster PTSD, psychological conditions, and student academic conditions. To address this gap, this study employed a questionnaire survey methodology in conjunction with a longitudinal tracking approach. Through comparative analysis and in-depth exploration, the study sought to analyze and explain the effect of post-disaster PTSD and the post-disaster psychological environment on students’ academic situations. The findings aim to offer educators and relevant stakeholders with empirical support to better understand the learning experiences of post-disaster students and facilitate the restoration of their academic standing.

The developmental stages including primary and secondary education represent a crucial period for the holistic growth of students, including both their physical and mental well-being. Cultivating positive academic emotions plays a critical role in cultivating the coordinated development of these emotions, thus contributing to the formation of a well-rounded personality and the enhancement of various skills. Therefore, prioritizing the establishment of a positive emotional environment in academic settings for primary and secondary school students holds paramount importance for their academic success, physical health, and mental health.

## 2 Material and method

### 2.1 Subjects

A longitudinal tracking methodology was employed to recruit participants from Guangling primary and secondary school in Weifang, Shandong Province, China, for a study involving three questionnaire surveys. The study’s participants were middle school students in grades 7 and 8, with ages ranging from 14 to 17 years old. The first survey (T1) was conducted 1 week following the disaster (September 2018) and yielded 195 valid questionnaires, with a gender distribution of 52.8% male and 47.2% female. Three months post-disaster (T2: December 2018), the second survey obtained 385 valid questionnaires, comprising 48.7% male and 51.3% female respondents. The final survey (T3), administered 1 year after the disaster (September 2019), resulted in 480 valid questionnaires. Of these, 195 participants were tracked from T1 and 263 from T2.

**Table:** 

Period	Variable	Sample analysis	Sample	Percentage	*N*
T1	Age	Male	103	52.80%	195
		Female	92	47.20%	
	Gender	14	24	12.35%	
		15	88	45.10%	
		16	78	40.0%	
		17	5	2.60%	
T2	Age	Male	187	48.70%	385
		Female	198	51.30%	
	Gender	11	39	10.12%	
		12	60	15.58%	
		13	88	22.86%	
		14	94	24.41%	
		15	61	15.84%	
		16	43	11.17%	
T3	Age	Male	128	48.70%	263
		Female	135	51.30%	
	Gender	11	27	10.20%	
		12	55	20.80%	
		13	71	26.9%	
		14	75	28.4%	
		15	34	12.9%	
		16	1	0.40%	

### 2.2 Methods

#### 2.2.1 Questionnaire survey

This study employed a multi-faceted survey instrument to collect data across four key domains. In the first part, demographic information was collected, including variables such as gender, grade level, age, and single-child status. The second part consisted of self-assessment survey, utilizing the Post-Traumatic Stress Disorder Checklist for Civilians (PCL-C) to evaluate the PTSD symptoms categorized into three dimensions, namely, “Repeated traumatic experience symptoms,” “emotional numbness and avoidance symptoms” and “irritability symptoms caused by excessive alertness” (Weathers, 1993). This instrument evaluates PTSD symptoms across three dimensions: re-experiencing symptoms, avoidance and emotional numbing symptoms, and hyperarousal symptoms. The PCL-C employs a 5-point Likert scale ranging from 1 (not at all) to 5 (extremely), with higher scores indicating a greater probability of PTSD. Further analysis of the PCL-C involved the study of three factors: intrusion (5 items), avoidance (7 items), and hyperarousal (5 items). Specifically, research by Yang et al. (2007) supports the reliability and validity of the PCL-C in the Chinese population. The third section of the survey assessed academic control utilizing the Adolescent Academic Control Scale developed by Perry et al. (2001). This scale comprises four items divided into two subscales: mathematics and English. Responses are offered on a 5-point Likert scale, with 1 indicating “strongly disagree” and 5 indicating “strongly agree,” where higher scores reflect a greater sense of control over academic performance. Finally, the Achievement Emotions Questionnaire (AEQ-M) was employed to measure academic emotions specifically related to mathematics learning (Pekrun et al., 2005). The AEQ-M comprises a set of 60 self-report items, each presenting a statement about one of seven mathematics-related emotions (two positive emotions, namely, enjoyment and pride; five negative emotions, namely, anger, anxiety, shame, hopelessness, and boredom) and asking students to indicate the degree to which that statement applies to them personally (Bieleke et al., 2023). The research by Bieleke et al. (2023) supports the structural properties of the AEQ-M correspond closely to predictions that can be derived from the control-value theory of achievement emotions, and these results are similar to those observed for achievement emotions in other school domains. Similar to the previous scales, the AEQ utilizes a 5-point Likert scale, with 1 representing “strongly disagree” and 5 representing “strongly agree” (Pekrun et al., 2005).

#### 2.2.2 Statistical methods

The analysis of the collected data was conducted utilizing the statistical software packages SPSS version 25.0 and Amos version 23.0. Following data entry, descriptive statistics were employed to characterize the post-disaster students’ PTSD symptoms, academic control, and academic emotions. Pearson correlation analysis was performed to assess the relationships between the variables and their dimensions. To further study the potential mediating role of academic control between PTSD and academic emotions, Amos software was employed. For all statistical analyses, a *p*-value of less than 0.05 was established as the threshold for statistical significance.

### 2.3 Procedure

Ethical approval for carrying out this study was obtained from a major research university in China. We contacted school administrators to explain our research purpose and asked for their permission to conduct the study. Teachers, students, and their parents were fully informed about the study objectives and the confidential nature of the study. Students’ assent and digital consents from their parents were obtained.

## 3 Results

### 3.1 Comparison of post-disaster PTSD and each dimension before and after

A statistically significant difference was observed in the total PTSD symptom scores and across all three symptom dimensions between T1 and T3 for the post-disaster students. Specifically, at T3, all three symptom dimensions exhibited a significant increase compared to T1, accompanied by a significant increase in the total PTSD score (see Table 1).

**Table 1:** Comparison of PTSD and each dimension at T1 and T3.

Dimension	T1 Mean ± SD	T3 Mean ± SD	*t* Value	T1 Detection rate	T3 Detection rate	*χ*^2^ Value
Re-experiencing symptoms	8.43 ± 3.23	10.43 ± 4.99	−5.84	9.7%	27.2%	18.09
Emotional numbing and avoidance symptoms	10.77 ± 4.52	12.78 ± 5.57	−5.54	10.8%	22.1%	33.38
Hyperarousal symptoms	9.10 ± 3.92	10.56 ± 4.69	−5.10	22.1%	36.9%	29.30
PTSD total score	28.30 ± 10.40	33.77 ± 13.60	−6.68	12.8%	28.2%	37.99

### 3.2 Analysis of the overall status of students’ academic control and academic emotions 3 months and 1 year after the disaster

The mean values of T2 and T3 were 15.28 and 15.46 respectively, indicating that students’ sense of control in mathematics was relatively high. In the same study of T2 and T3, happy academic emotion is higher than anxious academic emotion, indicating that students have better academic emotional experience (see Table 2).

**Table 2:** The mean and standard deviation of students’ academic control and academic emotion in the two measurements.

Dimension	T2 Mean ± SD	T3 Mean ± SD	Standard deviation
Mathematics academic control	15.28 ± 2.91	15.42 ± 3.09	2.91
Mathematics anxiety academic emotion	7.66 ± 2.86	9.17 ± 3.10	2.86
Mathematics pleasure academic emotion	15.77 ± 1.46	26.3 ± 14.65	3.46

### 3.3 Correlation analysis between post-disaster 1 week PTSD and 1 year academic control and academic emotions

The total value of PTSD and its three dimensions in T1 period were negatively correlated with the sense of academic control (T3). PTSD (T1) and its three dimensions were significantly negatively correlated with math pleasure academic emotion (T3), and significantly positively correlated with math anxiety academic emotion (T3). The sense of academic control (T3) was significantly positively correlated with mathematical pleasure academic emotion (T3) and negatively correlated with anxious academic emotion (T3) (see Table 3).

**Table 3:** Person correlation coefficients of PTSD (T1), students’ sense of academic control (T3), and academic mood (T3) after disaster.

	1	2	3	4	5	6	7
1.PTSD total value	1						
2.Repeated traumatic experience symptoms	0.857^**^	1					
3.Emotional Numbness and Avoidance Symptoms	0.910^**^	0.670^**^	1				
4.Irritability due to hypervigilance	0.894^**^	0.673^**^	0.707^**^	1			
5.Sense of academic control	−0.337^**^	−0.269^**^	−0.294^**^	−0.332^**^	1		
6.Anxiety Academic Emotions	0.341^**^	0.301^**^	0.283^**^	0.329^**^	0.579^**^	1	
7.Pleasant academic mood	−0.189^**^	−0.187^**^	−0.176^*^	−0.143^*^	0.493^**^	0.596^**^	1

### 3.4 Mediation analysis between PTSD 1 week post-disaster and academic control and academic emotions 1 year post-disaster

**Figure 1:**
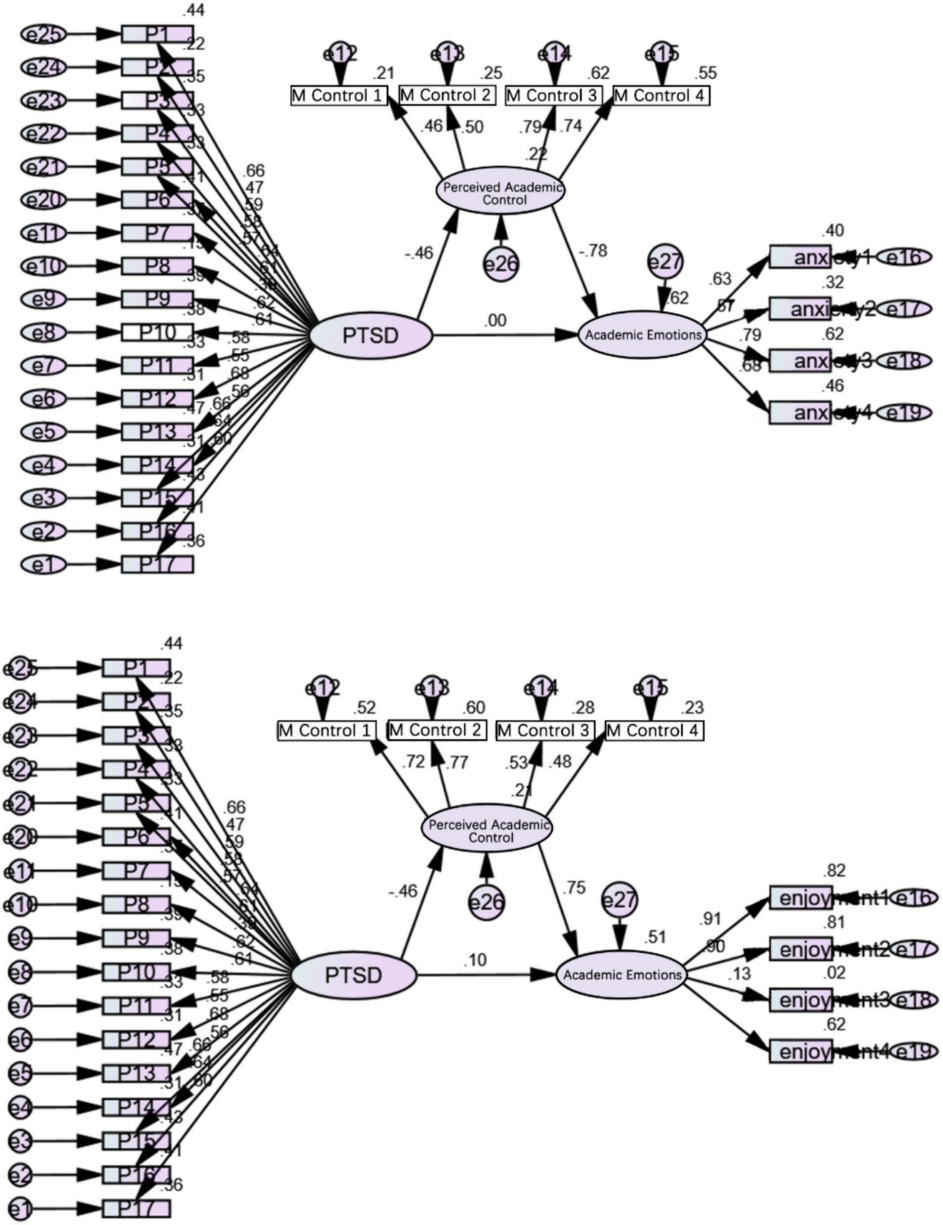
Standardized path coefficient diagram of structural equation model.

**Figure 2:**
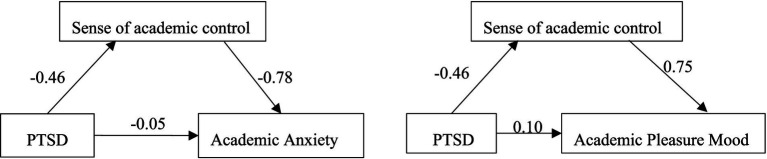
Mediation model of post-disaster 1-week PTSD between sense of academic control and academic mood 1 year after the disaster.

Figure 1 illustrates that in the absence of the mediating variable T3 mathematics academic control, students’ PTSD 1 week after the disaster can significantly positively predict T3 academic anxiety and negatively predict T3 academic pleasure. However, upon introducing academic control as a mediating variable, the negative predictive effect of PTSD 1 week after the disaster on T3 academic pleasure is no longer significant. These results offer evidence for the complete mediating role of T3 mathematics academic control (see Figure 2).

### 3.5 Correlation analysis between academic emotions and academic control variables at 3 months and 1 year after the disaster

There was a significant positive correlation between the sense of academic control in T2 and the sense of academic control in T3. The sense of academic control in the T2 period was significantly positively correlated with happy academic mood (T2 & T3), and the sense of academic control in the T3 period was significantly positively correlated with happy academic mood (T2 & T3) (see Table 4).

**Table 4:** Pearson correlation coefficients of students’ academic control (T2, T3) and academic emotions (T2, T3).

	1	2	3	4	5	6
1.Sense of academic control (T2)	1					
2.Sense of academic control (T3)	0.323^**^	1				
3.Anxiety about academic emotions (T2)	−0.312^**^	−0.263^**^	1			
4.Pleasant academic mood (T2)	0.487^**^	0.297^**^	−0.643^**^	1		
5.Anxiety about academic emotions (T3)	−0.266^**^	−0.540^**^	0.242^**^	−0.234^**^	1	
6.Pleasant academic mood (T3)	0.306^**^	0.538^**^	−0.250^**^	0.360^**^	−0.664^**^	1

### 3.6 Cross-lagged analysis of academic emotions and academic control at 3 months and 1 year post-disaster

**Figure 3:**
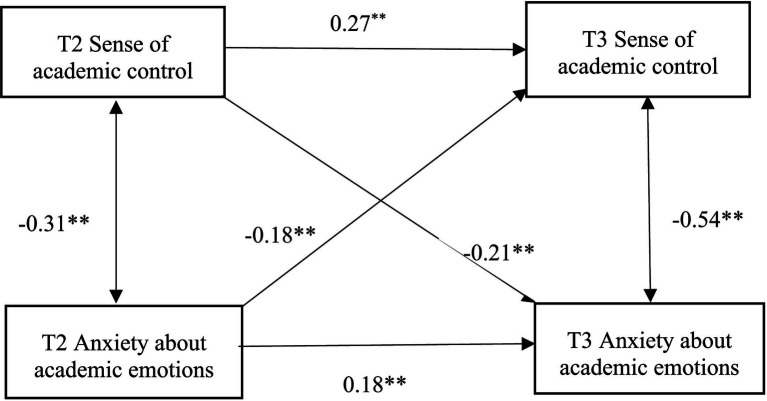
Mediation model of post-disaster 1-week PTSD between sense of academic control and academic emotion 1 year after the disaster. The two arrows in the figure represent the results of correlation analysis (correlation coefficient *r*), and the one-way arrows represent the results of regression analysis (standardized regression coefficient *β*), the same below.

**Figure 4:**
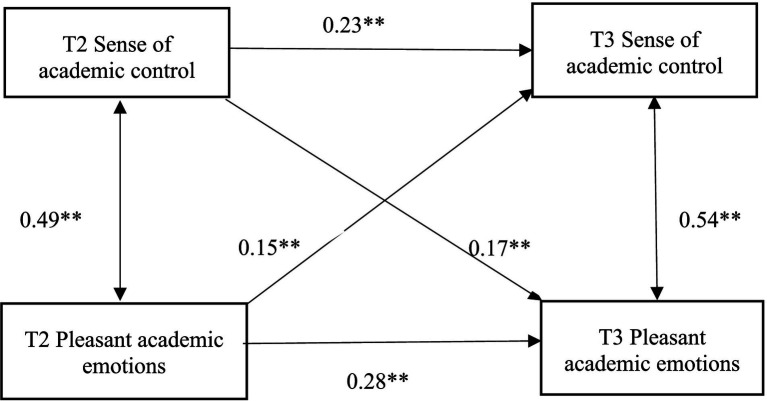
Cross-lagged analysis of academic sense of control and pleasant academic emotions.

Regression analysis indicated a significant causal relationship between the sample’s overall sense of academic control and their academic emotions, thereby supporting the interaction effect model initially proposed (see Figures 3, 4).

## 4 Discussion

The study’s findings indicated a significant prevalence of PTSD symptoms among the student population, with 12.8% exhibiting such symptoms 1 week following the disaster. This highlights the significant effect of the flood on the students’ mental well-being. Specifically, symptoms of irritability originating from post-disaster hypervigilance were significantly higher compared to those associated with re-experiencing the traumatic event and emotional numbing or avoidance behaviors. Such irritability symptoms are due to sleep disturbances, irritation, difficulty in concentrating, feelings of insecurity, and an increased startle response following exposure to a traumatic event. These conclusions align with the research conducted by Wu et al. (2015), which explored the mechanisms influencing post-traumatic psychological reactions in adolescents. The study hypothesizes that major natural disasters, such as floods, characterized by their sudden onset, short duration, and widespread devastation, contribute to feelings of helplessness and insecurity among students. This can trigger significant psychological distress, potentially leading to psychological trauma in certain cases. Moreover, the presence of PTSD has been associated with an increased incidence of sleep disturbances and a reduced quality of life.

To gain deeper insights into the progression of PTSD among students following a disaster, this study employed a longitudinal design. By comparing PTSD scores across three dimensions at 1 week and 1 year post-disaster, we discovered that both the overall detection rate of PTSD and its subscale scores were significantly higher at T3 than T1. Specifically, the detection rates of irritable symptoms, repeated trauma experience symptoms and emotional numbness avoidance symptoms caused by students’ over-alertness after disaster were higher in T3 than in T1, and the differences were significant. This finding aligns with previous research demonstrating the persistence of PTSD long after traumatic events. For instance, studies indicate prevalence rates of 18.8% at 3 months and 24.2% at 9 months post-disaster (Wang et al., 2000). Some studies even report high PTSD incidence many years later (Jin et al., 2014). The incidence of post-disaster PTSD is high, and most of them are long-term. In the post-disaster reconstruction work, disaster survivors are unable to face the grief of losing loved ones and rebuilding a new life, which often leads to the delay of PTSD. This study believes that the psychological problems caused by floods are long-term, and the floods not only affect the local area, but also have different degrees of impact on other areas that perceive the floods. Flood-related PTSD can lead to more behavioral problems that can affect a teenager’s academic performance.

However, analyses have also discovered potential decreases in occurrence and symptom severity (Lin et al., 2013; Wu et al., 2013). In comparison, this study reported that the detection rate and mean value of T3 PTSD were higher than that of T1, that is, the total positive rate of PTSD in middle school students in the disaster area was on the rise over time.

The observed difference could be attributed to two primary factors. Firstly, the time proximity of the time point in this study, conducted merely 1 week post-disaster, contrasts with previous earthquake-related PTSD studies that commenced data collection mostly 3 months after the event, with the first assessment occurring after 1 year. The increasing PTSD symptom trend observed from 1 week to 1 year post-disaster may be explained by the lack of recent exposure to flooding in Shouguang City. The abrupt nature of the flood event may have hindered the immediate development of stress responses among students, resulting in lower initial PTSD scores and detection rates compared to the later assessments. This aligns with the findings of Zhang et al. (2008), which indicate a tendency for PTSD to have a chronic and delayed onset, with a significant proportion of cases exhibiting symptoms persisting beyond 6 months and, in some instances, for several years. Secondly, the declining trend in overall PTSD prevalence among middle school students observed in previous earthquake studies may be attributed to the support offered by various societal sectors, the implementation of enhanced mental health education initiatives in schools, and the gradual attenuation of students’ emotional distress (Liu et al., 2011). It is plausible that the post-disaster psychological support and ongoing attention offered to students in this study area may have been insufficient. In addition, limitations in the study design, including time constraints and logistical factors, precluded conducting multiple analyses and timely follow-up assessments, thereby hindering the ability to discern the specific underlying reasons for the observed trends. This may constitute a deficiency of this study.

Moreover, the study indicated a lower mean score (3.7) on the mathematics subscale of the academic control sense measure in the subject group compared to the data reported by Perry et al. (2001). This discrepancy may be attributed to the comparatively lower academic control levels observed in primary and secondary school students following the disaster, compared to their college-aged counterparts. Several factors likely contribute to this phenomenon. Firstly, primary and secondary school students exhibit developmental immaturity across various domains, resulting in a reduced capacity to effectively utilize environmental resources and reduce adverse factors. Therefore, their ability to anticipate and regulate their own behaviors is also limited. Secondly, post-disaster PTSD can significantly affect students’ academic control, with sudden natural disasters further disrupting their academic control. Finally, the employed questionnaire may not comprehensively assess the academic control abilities of students domestically.

Extensive research has established that academic emotions consist of a wide spectrum of human emotional experiences. The findings of this study indicate that, following a flood disaster, students exhibited higher levels of pleasant emotions compared to anxiety scores across two separate assessments. Specifically, the mean scores for pleasant emotions surpassed the theoretical median, suggesting that students’ positive academic emotions outweighed their negative academic emotions during the post-disaster learning process. This observation aligns with the conclusions of numerous studies, indicating a generally positive state of overall academic emotions among students. A longitudinal comparison indicated a decline in pleasant academic emotions 1 year after the disaster, compared to the levels observed 3 months post-disaster. Conversely, anxious academic emotions exhibited an increase over the same timeframe. This shift may be attributed to the increased academic pressure and increased difficulty level associated with the third year of junior high school, leading to test anxiety and classroom anxiety. However, it is important to note that positive academic emotions remained predominant in general.

Pekrun’s (2006) control-value theory hypothesizes that academic control is reflected in both environmental and behavioral dimensions. The behavioral aspects consist of expectations related to behavioral control and outcome, while the environmental aspects primarily pertain to environmental-outcome expectations, which refer to outcomes that individuals experience without active effort. External environmental factors affect students’ academic emotions through the appraisal of control and value, thus impacting their motivation, learning strategies, and academic achievement (Pekrun, 2006). According to the theory of control value, when teenagers agree that the knowledge and skills they have learned are of great significance, they will be more likely to have the emotion and behavior of enjoying learning. On the contrary, the weaker the sense of subjective meaning, the easier it is to hate learning. This study demonstrates a significant association between perceived academic control and academic emotions, specifically a positive correlation with pleasant academic emotions and a negative correlation with anxious academic emotions. In addition, academic control at the three-month mark post-disaster predicted academic emotions 1 year later, and conversely, academic emotions at 3 months predicted academic control at the one-year mark. The control-value theory highlights academic control as a crucial antecedent of academic emotions. This theoretical framework hypothesizes that students’ accurate perception of opportunities or threats in the external environment, their perceived level of mastery in planning and regulating learning tasks, and the perceived value or usefulness of those tasks, constitute the main reasons of academic emotions.

Employing a cross-lagged analysis, this study indicated a reciprocal causal relationship between overall academic control and academic emotions in the sample. The findings indicate that academic control not only affects but also predicts academic emotions, while conversely, academic emotions affect academic control. Therefore, cultivating positive academic emotions during the learning process holds significant implications for enhancing students’ learning and development.

Analysis of the data indicates a significant correlation between PTSD symptoms 1 week following a disaster and academic control 1 year later. This aligns with Pekrun’s (2006) assertion that environmental factors can directly affect students’ academic control, emotions, and finally, their achievement. However, it is crucial to recognize that the environment itself is neutral; its effect is mediated by the individual’s sense of control. Students with a strong sense of academic control possess the resilience and determination to persevere even in the face of adversity, leading to positive academic performance. In addition, academic emotions play a crucial role in shaping students’ perceptions of their environment, primarily through the mediating variable of academic control. This model highlights the significance of an individual’s subjective interpretation of their environment, rather than the environment itself, in shaping their academic experiences.

Considering the interconnected nature of PTSD, academic control, and academic emotions in a mediation model, each factor holds the potential to affect the remaining two. Therefore, in the aftermath of a flood disaster, interventions aimed at reducing post-traumatic stress disorder in students, enhancing their sense of academic control, and cultivating positive academic emotions while reducing anxiety can be approached from various angles. These include improving students’ cognitive abilities and overall academic aptitude, enhancing their environmental evaluation skills, and cultivating an environment conducive to positive academic emotions. Finally, for the purpose of enhancing students’ academic performance, it is crucial to address the factors that affect their academic performance.

### 4.1 Shortcomings and prospects

First, this study employed a longitudinal research design, utilizing follow-up questionnaires administered at 1 week, 3 months, and 1 year post-disaster. While this design facilitated the study of temporal trends, it lacked a control group for comparative analysis. Future analyses could benefit from extending the follow-up period and increasing the frequency of data collection, while also incorporating a suitable control group to isolate the effects of the disaster. Besides, this study’s scope was limited to Guangling primary and secondary schools in Shouguang City, the disaster area. This geographical constraint restricted the ability to track all participants across all three data collection points, resulting in a reduced paired sample size. Expanding future research to consist of other provinces, regions, or post-disaster situations would enhance the generalizability and reliability of the findings. Finally, the reliance on student self-reports to assess PTSD, academic control, and academic emotions introduces the possibility of systematic bias. Future research could reduce this limitation by employing a multi-method approach, integrating interviews, observational methods, and physiological measures to offer a more comprehensive understanding of the mechanism of action between PTSD, academic control, and academic emotions. Concurrently, incorporating curriculum analysis, comparing pre- and post-disaster curricula, could offer valuable insights and enrich the existing body of knowledge in this field.

## 5 Conclusion

This study demonstrates that students display a range of PTSD symptoms following a flood disaster, with PTSD negatively impacting their academic standing. In addition, students demonstrate a reduced capacity for emotional regulation, hindering healthy personality development. In the work of alleviating PTSD symptoms, improving academic emotions, and enhancing academic control in post-disaster primary and secondary school students, efforts should prioritize the relationship between these factors. By cultivating a stronger sense of academic control and cultivating positive academic emotions, we can support better psychological adjustment and academic performance. This approach will facilitate augmented academic performance and contribute to the robust development of students’ personalities.

## Data availability statement

The original contributions presented in the study are included in the article/supplementary material, further inquiries can be directed to the corresponding author.

## Ethics statement

The studies involving humans were approved by Institutional Review Board of Psychology, Qingdao University. The studies were conducted in accordance with the local legislation and institutional requirements. Written informed consent for participation in this study was provided by the participants' legal guardians/next of kin.

## Author contributions

LZ: Writing – original draft. HW: Writing – original draft. KW: Writing – review & editing. CS: Writing – review & editing. MT: Writing – review & editing, Funding acquisition.
